# Money plant leaf (Epipremnum aureum): A comprehensive study of raw datasets with manual classification

**DOI:** 10.1016/j.dib.2025.112066

**Published:** 2025-09-15

**Authors:** Jameer kotwal, Aarju Jain, Ramgopal Kashyap, Anil Pise, Pramod Patil, Vinod Kimbahune

**Affiliations:** a Dr. D. Y. Patil Institute of Technology, Pune 411018, India; b Guru Ghasidas Vishwavidyalaya, Bilaspur 495009, India; c Senior Data Scientist - X-idian, Technical Partner- Cumulus Solutions, X-idian, Cumulus Solutions, Johannesburg, Gauteng 2057, South Africa

**Keywords:** Money plant leaf (epipremnum aureum), Datasets, Image classification, Machine learning, Deep learning

## Abstract

Money plants are widely recognized for their significant spiritual and air-purifying benefits. Research has proven that daily interaction with these vibrant indoor plants effectively reduces anxiety and stress. This paper introduces a robust dataset of **4302 healthy, unhealthy, combined, real and college premises** images of money plants captured using smartphones. The dataset **was** collected from the educational hub, Dr. D. Y. Patil Institute of Technology, Pune campus, Maharashtra, India. Under controlled conditions, images were taken from a mobile device to ensure consistency and quality. From different angles and different backgrounds, images are captured. The aim of creating the dataset **was** to support researchers in achieving their objectives in the agricultural field and to explore our dataset so that it may be used for further research, investigation, and training of artificial intelligence models using our dataset.

Specifications Table

**Table:** 

Subject	Agronomy
Specific subject area	**M**oney plant leaf disease, machine vision
Data format	Pre-processed and raw data
Type of data	Images of Money plant leaf
Data collection	**S**martphones are used to collect images. Itel S23, 50-megapixel dual camera
Data source location	Dr D. Y. Patil Institute of Technology, Pune, Maharashtra, India 411,018. 18.6231° N latitude, 73.8160° E longitude.
Data accessibility	**Repository name**: Epipremnum aureum (Money Plant Leaf) datasets **Data identification number**: Version: V4, 10.17632/kd8hs7ch6t.4 **Direct URL to data:**https://data.mendeley.com/datasets/kd8hs7ch6t/4 **Zenodo Link:**https://zenodo.org/records/14598223 **GitHub Link:**https://github.com/jameerktwl/Plant-Leaf-disease-detectionandn-classification-using-EfficientNet-and-UNet 10.5281/zenodo.14598222
Related research article	**Case study:** **Article Title:** Enhanced leaf disease detection: UNet for segmentation and optimized EfficientNet for disease classificationAuthor: Dr.Jameer Kotwal, Dr.Ramgopal Kashyap, Dr.Shafi Pathan**Paper Link:**https://www.sciencedirect.com/science/article/pii/S2665963824000897 [1] **Journal name:** Software Impacts

## 1 Value of the Data

• Scholars and data analysts may leverage this dataset to design and refine machine learning algorithms aimed at categorizing leaves as either healthy or unhealthy. Such initiatives could culminate in the development of automated and precise instruments for assessing plant health [[2], [3],^4^].
• The dataset presents avenues for computer vision scholars to investigate novel methodologies in pattern recognition while improving classification strategies for medicinal plant taxa [[5], [6], [7]].
• The dataset functions as a significant pedagogical instrument for **programmers** in segmentation and computer vision. It enables researchers to engage with authentic datasets, thereby enhancing experiential learning and the cultivation of practical competencies in these fields [8].
• This dataset is vital for research in the agriculture field, and with the help of computer vision algorithms, to keep surveillance of plant vitality, diagnose at an incipient stage, and endorse sustainable agricultural practices.

## 2 Purpose of collecting the dataset

• **Academic & Educational Purpose**
  - The dataset is useful for students and academics to learn more about data analysis techniques and the case study.
• **Research Analysis:**
  - Researchers collect data to validate and to prove or disprove hypotheses in various fields [9].
  - Use data to contribute to a broader understanding of a field and analyse trends, patterns, and co-relationships within the data.
• **Model development & Training:**
  - Collecting labelled or unlabelled data to train and test machine learning models for prediction, classification, clustering, etc.
  - Check the performance of algorithms and improve their accuracy [10].
• **Monitoring and Evaluation:**
  - To ensure quality, the standard of data, and to track progress towards specific goals.

## 3 Background

The money plant (Epipremnum aureum), commonly referred to as Devil's Ivy, Golden Pothos, or Scindapsus aureus, is a widely esteemed houseplant recognized for its remarkable resilience and aesthetically pleasing, cordate foliage. The money plant is indigenous to the Solomon Islands, Moorea, and various regions of Southeast Asia, including the Philippines, Indonesia, and Malaysia. In its natural habitat, it flourishes as a climbing vine within tropical forests, predominantly developing in the shaded environments beneath larger arboreal species. It skillfully climbs or sprawls along the ground, securely anchoring itself to tree trunks and structures with its aerial roots. Renowned for its glossy leaves, the money plant showcases a captivating variety of colors. Some cultivars display deep green foliage, while others are beautifully variegated with striking yellow or white streaks. Its rapid growth rate makes it a fantastic choice for hanging baskets, containers, or trellises, adding vibrant greenery to any space. Moreover, the money plant is celebrated for its impressive ability to enhance indoor air quality by effectively eliminating harmful substances like formaldehyde, benzene, and xylene. This air-purifying property solidifies its status as a **favorite** in both homes and professional environments.

Guiding Questions:

a) What is the age of the plants?
b) What are the cultivation procedures?
c) When were photographs captured?
d) How was classification performed?
e) What criteria were used for classification**?**

**Fig. 1:**
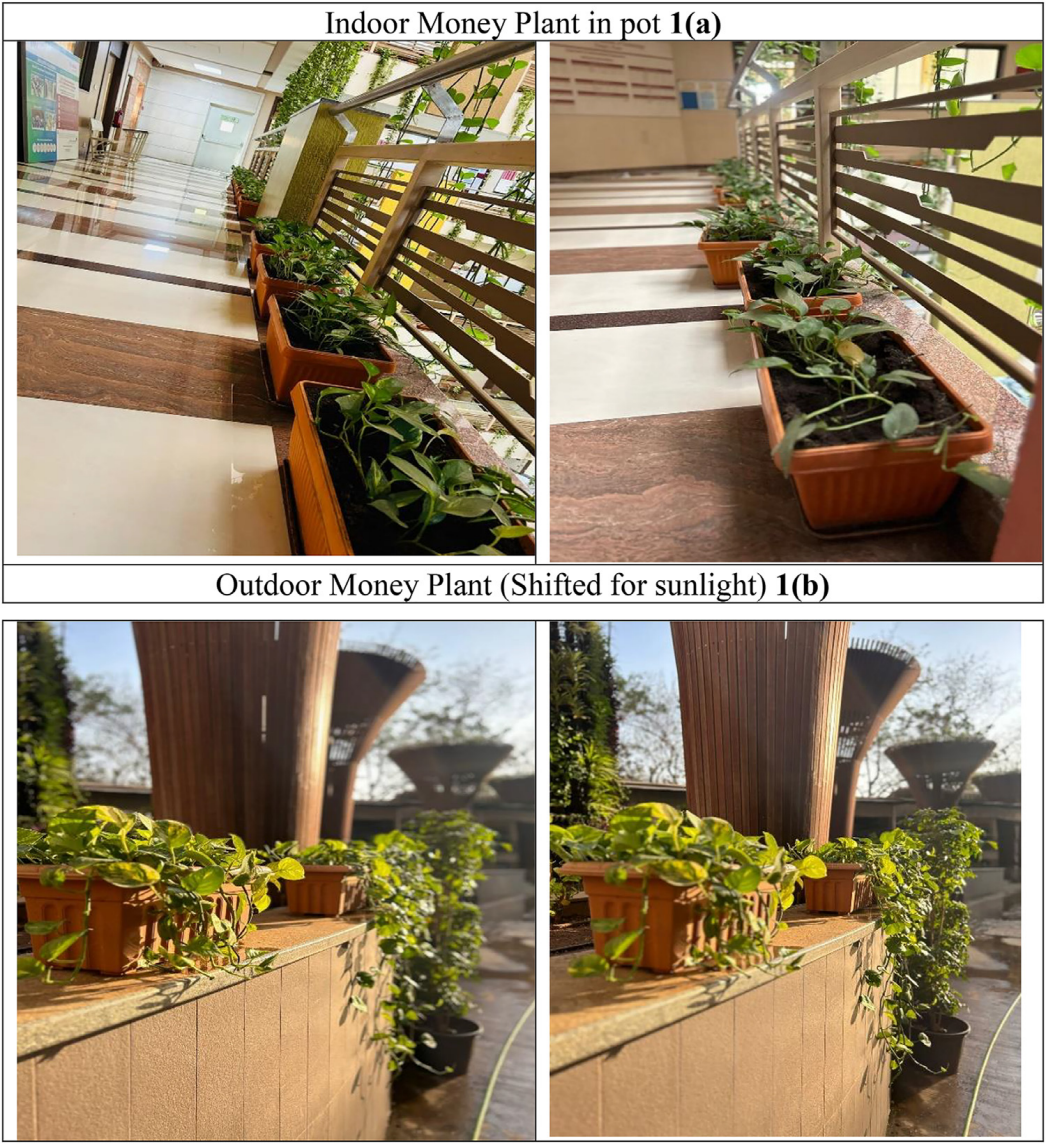
Indoor and Outdoor area.
c) Photographs are captured using smartphones. Usually, the photographs are captured in natural light to avoid direct sunlight and shadows on the leaves.
d) The money plant classification is performed with the help of experts from an agricultural background.
e) Based on leaf size, shape, color, texture, and mainly the health performance indicators like curly dry leaf, spots of diseases, color change**,** etc., are the criteria used to perform classification whether the leaf is healthy or not.

Based on the above guiding questions, the Money Plant leaf age, cultivation procedures, image captures**,** etc., are as:

a) The age of the money plant leaf is 8 to 24 months.
b) The plant’s strong stems are cut and placed in soil. Once the plant outgrows its pot, repot it into a larger one. For every 4 to 6 months, the money plant pot is shifted outdoors for sunlight. After staying outside for 10 to 15 days again the plants are repotted too indoors **as shown in** Fig. 1(a) and (b).

### 3.1 Objectives

a) The money plant dataset is used to classify the healthy and unhealthy leaf using an **artificial intelligence** algorithm in real time. **It is** a medicinal plant helpful for daily life.
b) The **Machine Learning** model performs well on pre-processed images, and the better the accuracy, the model can perform.

## 4 Data Description

The Money Plant Leaf dataset is a powerful resource designed to advance the creation, evaluation, and refinement of highly accurate machine learning models. Sourced from the esteemed Dr D. Y. Patil Institute of Technology in Pimpri, Pune, India, this comprehensive dataset is essential for conducting in-depth condition analysis research. With a strong focus on leaf quality assessment, it is precisely tailored for applications in computer vision and machine learning, making it a critical asset in the agricultural sector for plant identification, detection, classification, and health evaluation.

**Fig. 2:**
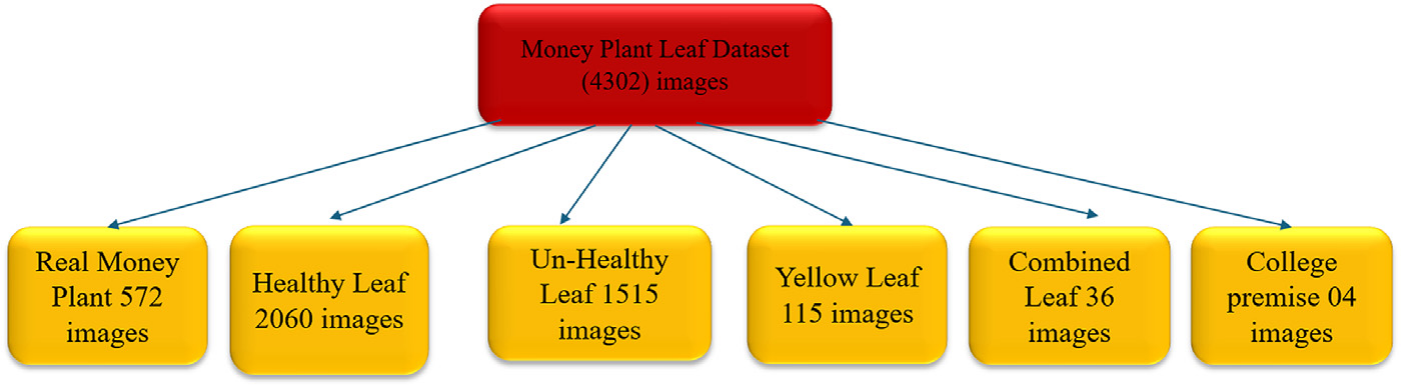
Dataset folder structure and count.

This dataset features two distinct classifications: healthy and unhealthy leaves. It includes an impressive collection of 2060 images of healthy leaves, 1515 images of unhealthy leaves, 115 images of yellow leaves, college premise 04 images, real money plants 572 images and 36 images showcasing both healthy and unhealthy leaves in a single frame. Furthermore, it contains a variety of images related to the college premises and real-world collections. Fig. 2 displays the folder structure of the Money Plant dataset along with the image counts, while Table 1 provides a detailed overview of the types of diseases represented and the number of images associated with each category.

**Table 1:** Types of disease and number of images.

Category	Number of images
**College Premises**	**4**
Healthy leaf	2060
Un-Healthy leaf	1515
Yellow leaf	115
**Combined leaf**	**36**
**Real Image Money Plant**	**572**
**Total**	**4302**

**Fig. 3:**
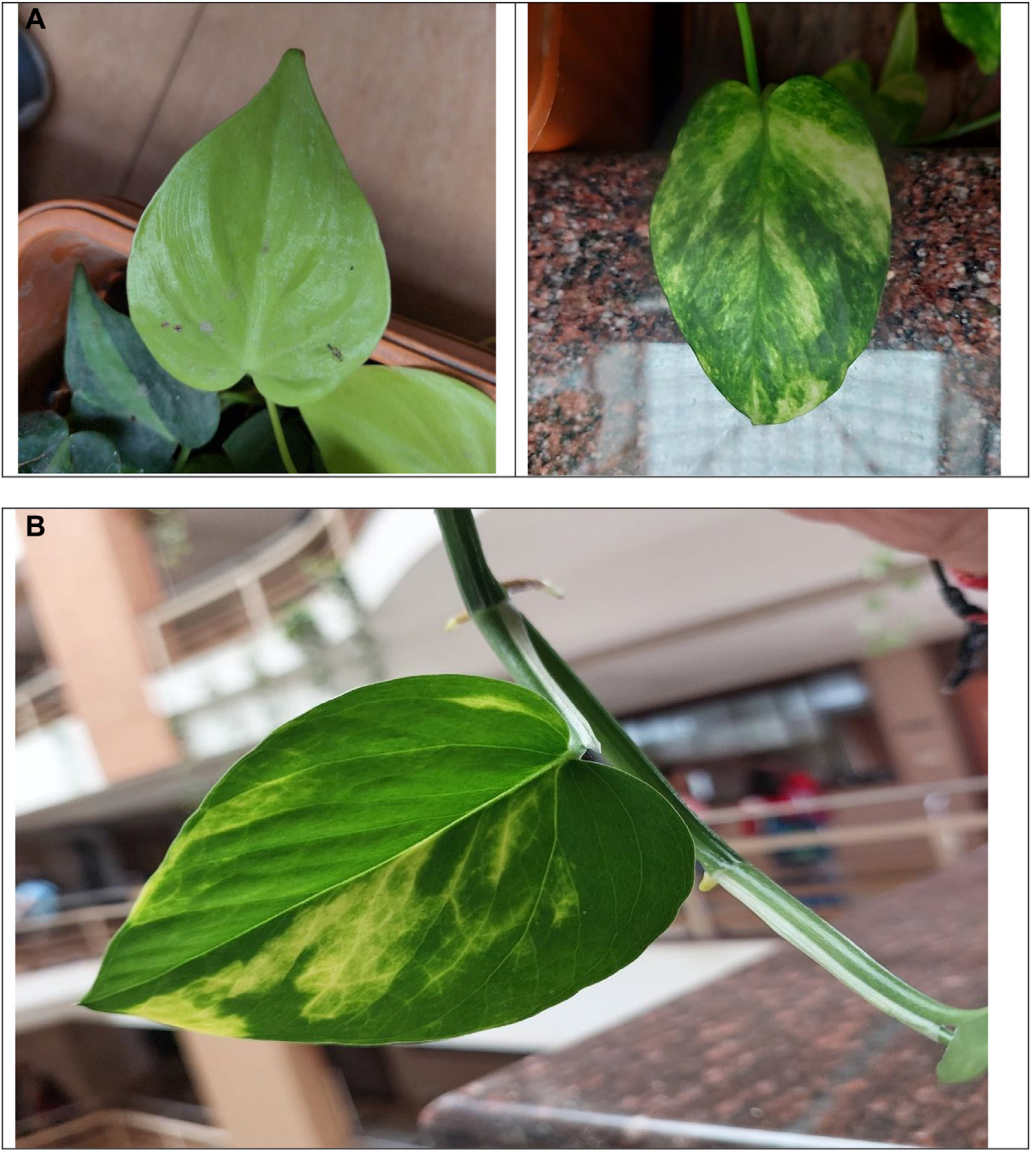
Healthy leaf image.

In this section, we analyse the atypical manifestations of various pathologies discerned within the leaf photographs of our dataset. Illustrations of each condition, alongside the control group, healthy leaf, are presented in Fig. 3.

Fungal, viral, and bacterial infections—primarily driven by environmental stress—are the major culprits behind Septoria brown spots, vein necrosis, and bacterial spots on the leaves of the money plant (Epipremnum aureum). While these problems are relatively rare in indoor plants, certain conditions, such as excess moisture, inadequate air circulation, and persistently wet leaves, can lead to Septoria brown spots, characterized by small brown patches with dark edges. Vein necrosis significantly weakens the plant and is almost always caused by viral infections, nutritional deficiencies (particularly a lack of potassium or magnesium), or water stress. Bacterial spots are present as water-soaked lesions, sometimes surrounded by yellow halos, typically because of bacteria like Xanthomonas or Pseudomonas, which thrive in humid conditions.

The likelihood of these diseases increases with factors such as overwatering, poor ventilation, contaminated water, and proximity to diseased plants. To effectively prevent these issues, it is crucial to ensure good airflow, avoid overhead watering, guarantee proper soil drainage, and swiftly remove any affected leaves. With consistent care, including balanced fertilization and regular application of neem oil, you can maintain a healthy, vibrant money plant that remains free from infections.

**Fig. 4:**
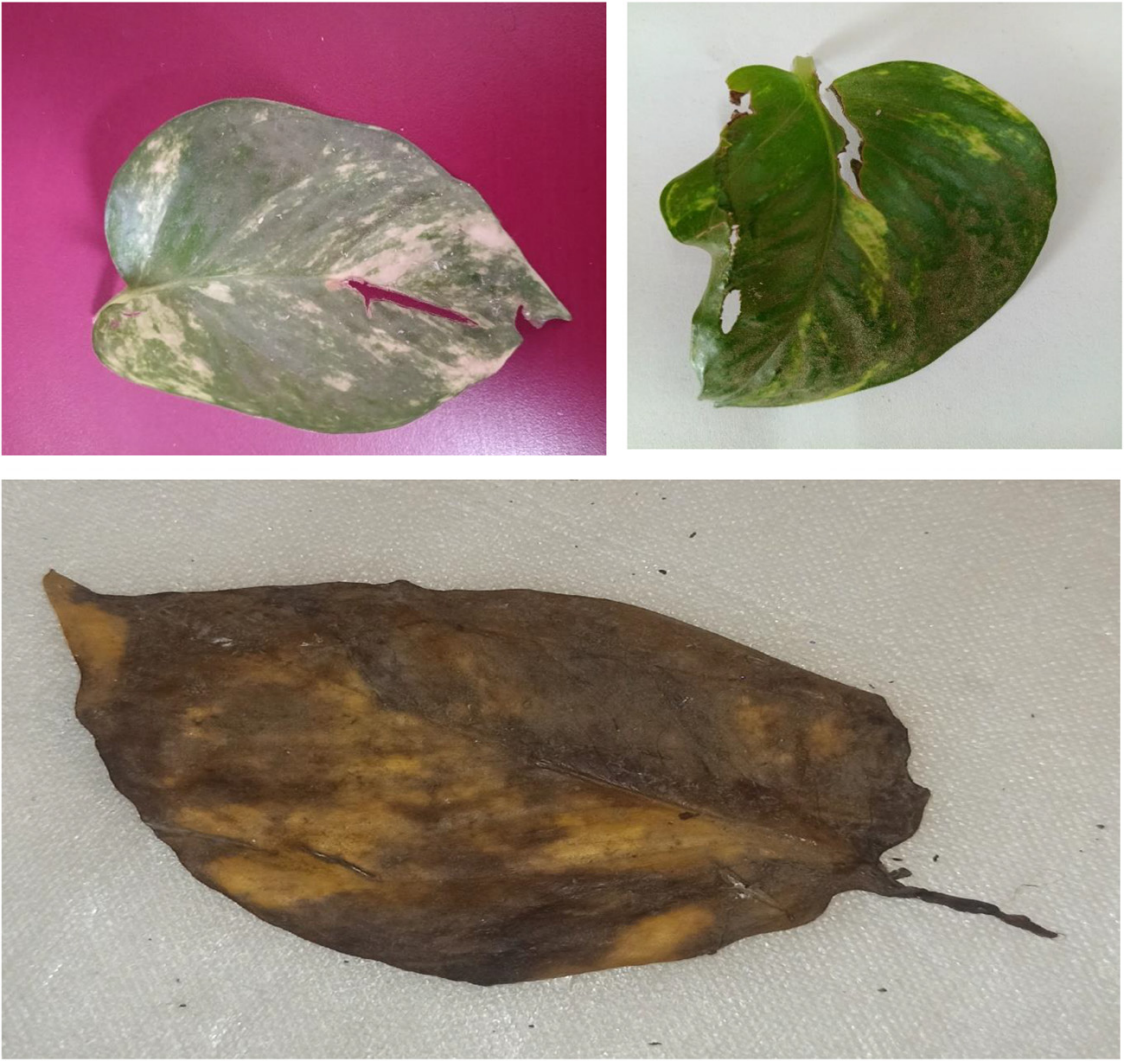
Unhealthy leaf image.

The common diseases that affect the leaves, such as Septoria brown spots, vein necrosis, and bacterial spots, are all categorized as indications of unhealthy leaves. Fig. 4 clearly illustrates an unhealthy leaf [[11], [12], [13]].

**Fig. 5:**
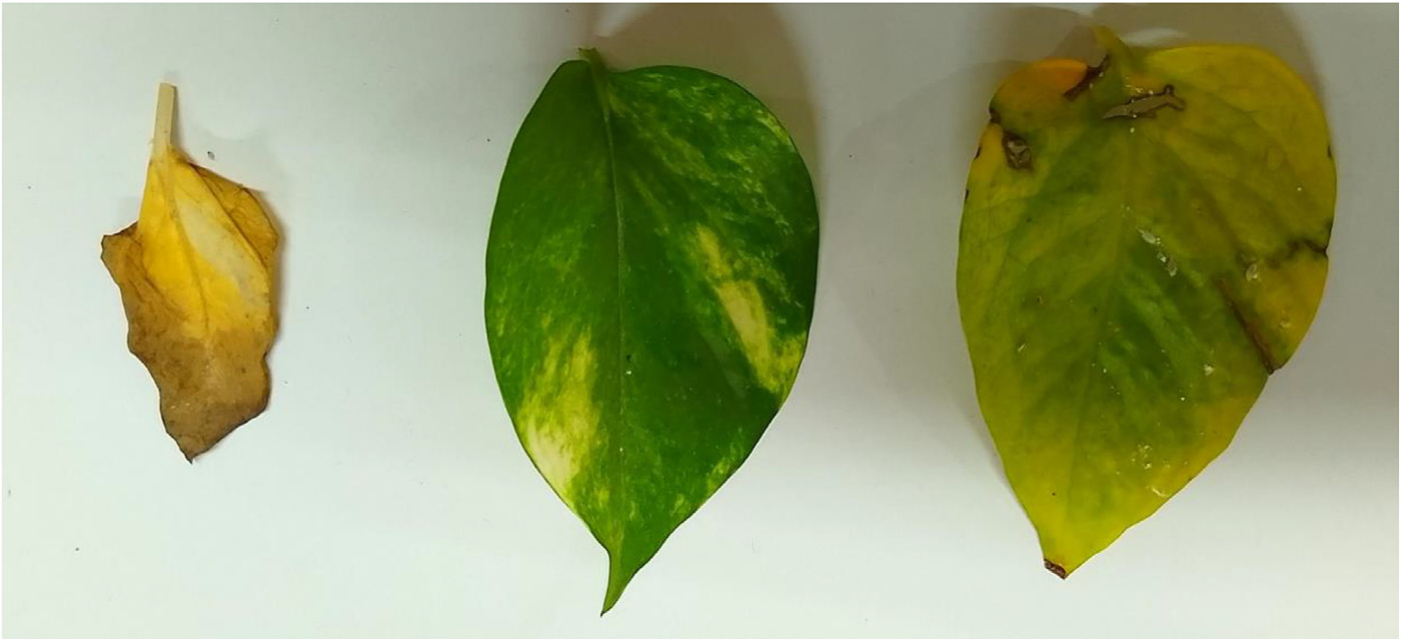
Combined image of a Healthy and Unhealthy leaf.

Fig.5 shows the combined image of healthy and unhealthy images so that the algorithm can predict in the same image which leaf is healthy and unhealthy.

**Fig. 6:**
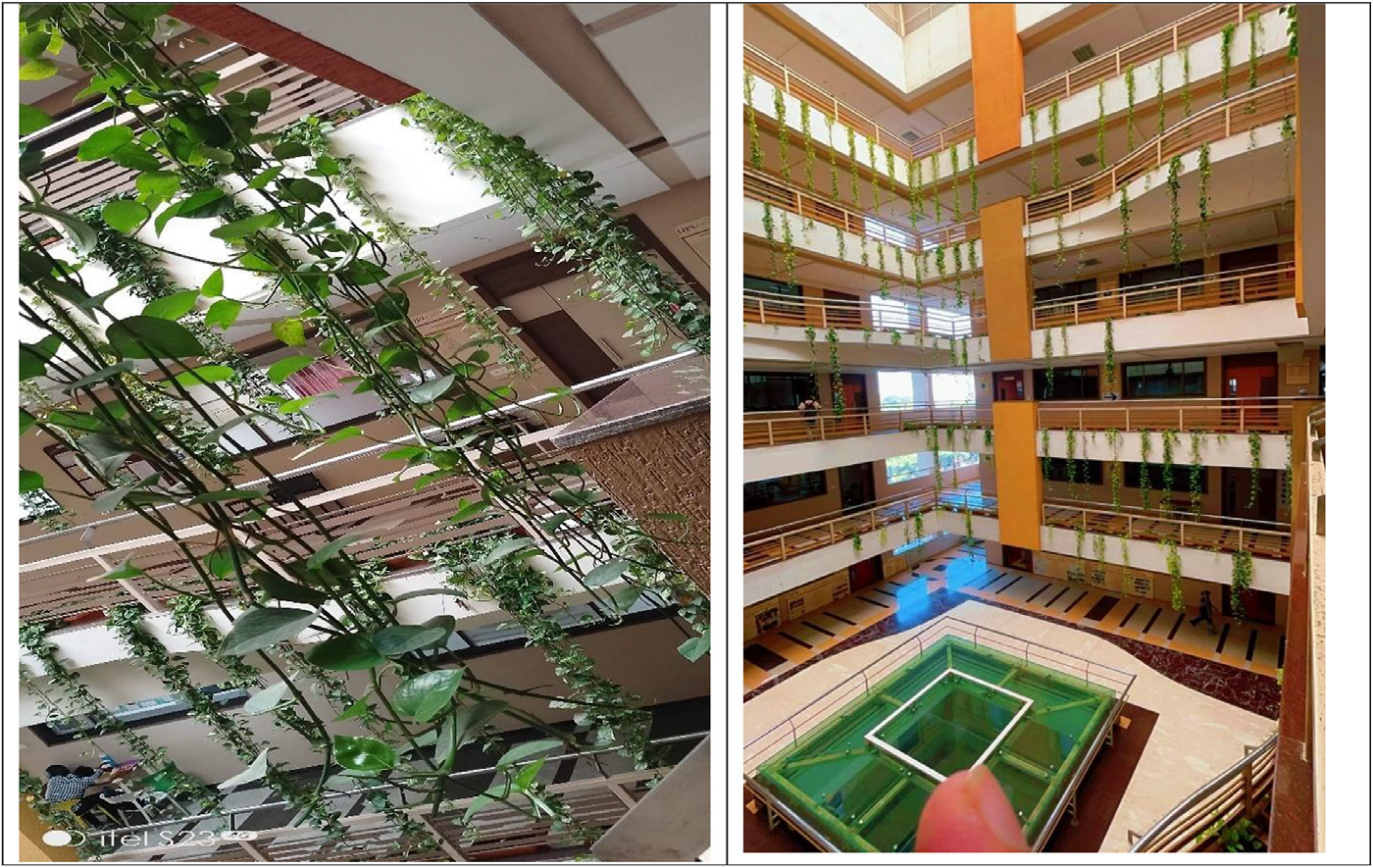
Money plant in the college building.

The college building consists of three blocks block A, B, and C. Each with the same structure, and a money plant is placed in every block and on each floor. Fig. 6 shows the college building.

## 5 Experimental Design, Materials, and Methods

Images are collected from Dr. D. Y. Patil Institute of Technology and captured from smartphones in the month of June to March in 2025.

**Fig. 7:**
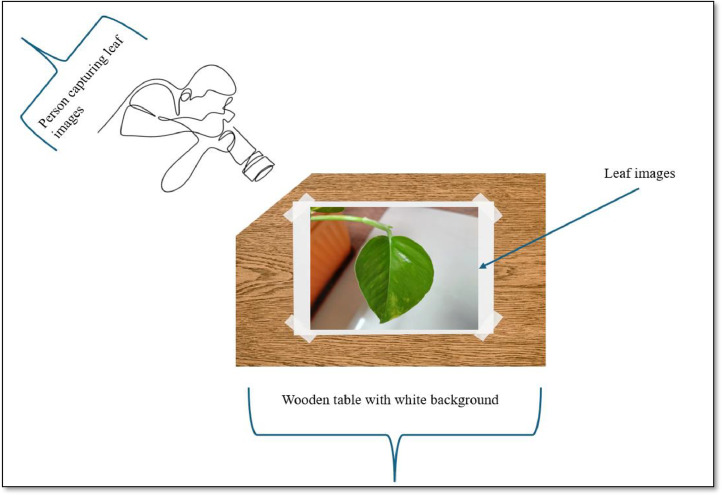
Experimental setup of dataset.
2) Image size
  The images in the Money Plant Leaf dataset are standardised to a resolution of 224×224 pixels. **The images are collected through smartphones; the size of the images is not the same, rather images are resized during the preprocessing stage.** This size ensures compatibility with most deep learning frameworks and pre-trained models, facilitating efficient processing and analysis. The resolution is carefully chosen to strike a balance between computational efficiency and sufficient detail for accurate classification and feature extraction.
3) Dataset split
  The Money Plant Leaf dataset is divided into two subsets to support model training and testing: **Training Set (80** **%)**: This subset is used to train machine learning and deep learning models by enabling them to learn patterns and features from the data.
  **Test Set (20** **%)**: This subset is reserved for final performance evaluation, ensuring the model's generalization capability on completely unseen data.

The photo pre-processing involved three key stages:

1) Augmentation
  The Money Plant Leaf dataset was collected at Dr D. Y. Patil Institute of Technology, Pimpri, Pune, India. The data acquisition process involved gathering high-quality images of money plant leaves under controlled environmental conditions to ensure consistency and reliability. Various factors such as lighting, angle, and background were carefully standardised to minimise noise and enhance the quality of the dataset. The images represent a diverse range of leaf conditions, including variations in health, size, texture, and appearance, to provide a robust foundation for machine learning and computer vision research. Fig. 7 contains the experimental setup for capturing the leaf image through smartphones.

## Limitations

The dataset **was** collected from a specific location and area of Dr. D. Y. Patil Institute of Technology. As a result of the variability in image quality stemming from environmental conditions and lighting, the consistency of models may be compromised in practical applications.

## Ethics Statement

We haven’t conducted any experiments on humans and animals.

## CRediT Author Statement

**Dr. Jameer Kotwal:** Conceptualization, Investigation, Software, Writing – original draft, first draft, Writing: review and editing. **Aarju Jain:** Resources, Conceptualization. **Dr. Ramgopal Kashyap:** Validation, Software, data curation, Writing – original draft, first draft. **Dr. Pramod Patil**: Supervision, Data Curation. **Dr. Vinod Kimbahune:** Methodology, writing, review, and editing. **Dr. Anil Pise:** formal analysis, Writing – original draft, first draft.

## Data Availability

Mendeley DataEpipremnum aureum (Money Plant Leaf) datasets (Original data).
